# Recent advances in the management of acromegaly

**DOI:** 10.12688/f1000research.7043.1

**Published:** 2015-12-11

**Authors:** Georgia Ntali, Niki Karavitaki

**Affiliations:** 1 Department of Endocrinology and Diabetes, Alexandra Hospital, 80 Vas. Sofias St, Athens, 11528, Greece; 2 Centre for Endocrinology, Diabetes and Metabolism, School of Clinical and Experimental Medicine, University of Birmingham, Wolfson Drive, Edgbaston, Birmingham, B15 2TT, UK

**Keywords:** Acromegaly, management, somatostatin analogues, adenomectomy, radiotherapy

## Abstract

Acromegaly is a rare condition of GH excess associated with significant morbidities (e.g. hypertension, glucose intolerance or diabetes mellitus, cardiac, cerebrovascular, respiratory disease and arthritis) and, when uncontrolled, high mortality. Surgery, medical treatment and radiotherapy remain our therapeutic tools. Advances in these options during the last years have offered further perspectives in the management of patients and particularly those with challenging tumours; the impact of these on the long-term morbidity and mortality remains to be assessed.

## Introduction

Acromegaly is a rare condition characterized by growth hormone (GH) excess and elevated insulin growth factor 1 (IGF-1) levels attributed in the vast majority of cases to a pituitary adenoma. Mortality is high in uncontrolled disease (standardized mortality ratio [SMR]: 0.94–2.5) ^1– 3^, but adequate biochemical control can restore it to normal (SMR: 0.44–1.13) ^1– 3^. Early diagnosis and treatment, close monitoring, and control of co-morbidities play a major role in the optimal management of these patients. Based on the recently published Endocrine Society Clinical Practice Guideline on acromegaly, therapeutic goals include an age-normalized serum IGF-1 value and a random GH level <1 μg/L ^4^. Surgery, drugs, and radiotherapy remain the mainstay of treatment, and this review will focus on recent advances in the management of this challenging condition.

## Surgery

In the last decade, endoscopic endonasal surgery, by offering improved visualization and less nasal trauma, is more widely utilized, replacing the traditional microscopic technique. Based on the 2010 consensus criteria, remission rates after endoscopic transsphenoidal surgery range between 63 and 100% for microadenomas ^5– 10^ and between 40 and 72% for macroadenomas ^5– 10^; these are comparable to those reported with microsurgical approaches. Peri-operative complications are also similar, apart from sinusitis and alterations in taste or smell, which are described more often in patients managed endoscopically ^7, 11^.

## Medical therapy

### Somatostatin analogues

Somatostatin downregulates GH secretion and induces cell cycle arrest and apoptosis through binding to five subtypes (somatostatin receptors 1–5 [SSTR1-SSTR5]) of G-protein-coupled receptors expressed on the somatotroph cells. Somatotroph adenomas express somatostatin receptor subtype 2 (SSTR2) and SSTR5 at high levels, and somatostatin analogues (SSAs) with greater specificity for these receptors and a longer half-life (than the 2–3 minutes of the natural somatostatin molecule) have been developed (octreotide and lanreotide).

***Somatostatin analogues as pre-operative medical therapy.*** Acromegalic patients present a challenge peri-operatively, as their airway anatomy and cardiovascular co-morbidities increase the risk of anesthetic complications ^12, 13^. Pre-operative treatment with SSAs is not recommended routinely ^4^, but in selected cases it may rapidly reduce soft tissue swelling, improve sleep apnea and cardiac function, and reduce intubation-related complications ^13– 16^. A recent meta-analysis of five retrospective controlled, two prospective non-randomized, and three prospective randomized controlled studies explored the role of pre-operative use of SSAs in improving biochemical cure rate after surgery; the results were consistent with a borderline significant effect (pooled odds ratio [OR] 1.62; 95% confidence interval [CI] 0.93–2.82). When only the three randomized prospective controlled trials were analyzed, a significant benefit was found with a pooled OR of 3.62 (95% CI 1.88–6.96) ^17^.

In a group of 30 newly diagnosed acromegalics, pre-surgical treatment with lanreotide autogel for 24 weeks induced tumor shrinkage ≥20% in 79% (23/29) and resulted in mean GH <1 μg/L and IGF-1 normalization in 33.3% (10/33) of the patients. Metabolic profile including fasting blood glucose, HbA1c, lipids, and blood pressure did not change significantly, but amelioration of arterial stiffness and endothelial function were documented. Notably, the apnea/hypopnea index improved in 61%, remained unchanged in 8.7%, and deteriorated significantly in 30.4% of the patients ^18^.

Fougner *et al.* evaluated the impact of pre-operative octreotide treatment on long-term remission. When both remission criteria of IGF-1 levels ≤ upper limit of normal (ULN) and nadir GH ≤2 mU/L on the oral glucose tolerance test were applied, no beneficial effect was confirmed 1 and 5 years post-operatively for both microadenoma and macroadenoma subgroups ^19^.

***Somatostatin analogues as primary therapy.*** The 2014 Endocrine Society Clinical Practice Guideline recommends that primary therapy with SSAs is principally used for a subgroup of patients with larger tumors when surgical cure is unlikely and, additionally, if surgery is refused or contraindicated ^4^.

Primary therapy with lanreotide autogel was evaluated in a prospective 48-week multicenter study which recruited 90 naive acromegalics with macroadenomas. Tumor shrinkage ≥20% was observed in 54.1%, 56.3%, and 62.9% of patients at 12, 24, and 48 weeks, respectively, and mean GH ≤1.0 μg/L and IGF-1 normalization was reported in 21.4%, 23.4%, and 30.6% of patients at the same time intervals ^20^.

Furthermore, a meta-analysis of 35 studies on treatment-naive acromegalics showed that in comparison with medical treatment, surgery was associated with higher remission rates at longer follow-up periods (≥24 months) but not at shorter follow-up intervals (≤6 months) ^21^.

The effect of different treatment modalities on mortality rates was evaluated in 438 acromegalic patients for the period 1966–2009 by Bogazzi *et al.* ^22^. Interestingly, after correction for several covariates, the risk of death in patients receiving primary SSA therapy was five times higher than the risk in all patients submitted to adenomectomy (hazard ratio [HR]: 5.52, 95% CI 1.06–28.77, p=0.043). Stratification of patients by the presence of diabetes mellitus revealed that diabetic patients receiving primary SSA therapy had a significantly elevated HR of 21.94 (95% CI 1.56–309.04, p=0.022), whereas in non-diabetic ones the HR was only 1.30 (95% CI 0.04–38.09, p=0.881) ^22^.

***Somatostatin analogues as adjuvant medical therapy.*** SSAs are a treatment option for patients with significant persistent disease after surgery ^4^. Adenoma GH granularity and SSTR2A-positive immunohistochemistry may predict the response with the densely granulated and those expressing SSTR2A being more responsive ^23– 27^. Sparsely granulated adenomas exhibit lower SSTR2 expression and respond less to somatostatin receptor ligands (SRLs) ^25, 27^; it is presumed that, if SSTR5 expression is present, response to novel SSAs may be the case ^28^. It has also been reported that Ki-67 is higher in non-SSA responders ^29, 30^.

T2 intensity on magnetic resonance imaging (MRI) has also been proposed as a marker of responsiveness. Hypointense T2-weighted MRI signal is associated with a better responsiveness to octreotide than an isointense or hyperintense one in patients not cured after surgery ^31^. Notably, it has been suggested that hyperintense tumors have lower baseline GH and IGF-1 levels and tend to be larger than the hypointense ones. T2 intensity and granulation pattern correlate and sparsely granulated adenomas tend to be hyperintense ^32, 33^.

***Effectiveness of somatostatin analogues.*** A recent meta-analysis on the biochemical response rates to SSAs as primary or adjuvant therapy – including retrospective and prospective studies with both short- and long-acting octreotide and lanreotide formulations (sustained-release and depot/autogel) – between 1987 and 2012 (total of 3787 patients) showed that the average GH control rate was 56% and the IGF-1 normalization rate was 55% with no significant difference in the effectiveness between the different SSA agents ^34^.

Furthermore, a meta-analysis of 41 studies on the effects of octreotide (as first-line or adjuvant therapy) on tumor shrinkage revealed that overall 57% of patients achieved >20% volume decrease. Tumor shrinkage was greater in patients treated with octreotide long-acting release (LAR) rather than subcutaneous octreotide and in those achieving mean GH levels below 2.0–2.5 ng/ml or normal IGF-1 ^35^.

The option of discontinuing octreotide in patients well-controlled on low doses offered at long intervals was studied in 12 responders fulfilling the following criteria: single basal GH <1.5 ng/ml and IGF-1 <1.2*ULN, no history of irradiation or recent dopamine agonist use, and tumor remnant <8 mm after discontinuation of octreotide. Five patients (41.7%) remained in remission after 12 months of follow-up and seven (58.3%) relapsed within 1 year of discontinuation. Interestingly, three out of five subjects who remained in remission were on octreotide LAR injections every 12 weeks but none of the seven who recurred ^36^.

Finally, a number of studies have shown that surgical debulking of the adenoma improves the subsequent response to SSAs ^37– 39^.

**Oral octreotide**

Recently, oral octreotide capsules (OOCs) have become available. The capsule contains 20 mg non-modified octreotide acetate formulated with a novel transient permeability enhancer, which enables transient and reversible paracellular tight junction passage of molecules <70 kDa. This new formulation facilitates intestinal octreotide absorption.

OOC effectiveness in maintaining biochemical response to injectable SSAs has been assessed in a phase III multicenter, open-label, dose-titration, baseline-controlled study. A total of 155 complete or partially controlled patients (IGF-1 <1.3*ULN, and 2-h integrated GH <2.5 ng/mL) receiving injectable SSAs for ≥3 months were switched to 40 mg daily OOCs; the dose was escalated to 60 mg and then up to 80 mg daily. Subsequent fixed doses were maintained for a 7-month core treatment, followed by a voluntary 6-month extension. A total of 65% of the participants maintained response and achieved the primary endpoint of IGF-1 <1.3*ULN and mean GH <2.5 ng/mL at the end of the core treatment period; this rate was 62% after 13 months’ treatment ^40^. Further studies on the efficacy, safety, and convenience of this formulation are needed.

**Pasireotide**

Pasireotide is a novel multi-ligand SRL with high affinity for SSTR1, SSTR2, SSTR3, and particularly SSTR5. In a head-to-head comparison prospective study of pasireotide *vs.* octreotide in 358 medical treatment-naive patients, biochemical control (defined as mean GH <2.5 μg/L and normal for age IGF-1) at 12 months was achieved in a higher percentage of patients on pasireotide in both the *de novo* and the post-surgical groups (31.3% *vs.* 19.2 *%; p*=0.007). Interestingly, the two drugs had the same effect on GH inhibition, but pasireotide was more effective in reducing IGF-1 ^41^.

The PAOLA study randomly assigned 198 inadequately controlled patients (previously treated with 30 mg octreotide long-acting or 120 mg lanreotide autogel as monotherapy for 6 months or longer) to pasireotide 40 mg, pasireotide 60 mg, or continued treatment with octreotide or lanreotide. At 24 weeks, 15% of the patients of the pasireotide 40 mg group and 20% of the pasireotide 60 mg group achieved biochemical control; no patient in the sustained octreotide or lanreotide group achieved this goal (absolute difference from control group 15.4%, 95% CI 7.6–26.5, *p*=0.0006 for pasireotide 40 mg group, 20.0%, 95% CI 11.1–31.8, *p*<0.0001 for pasireotide 60 mg group) ^42^.

It should be noted that the impact of pasireotide on blood glucose control remains a concern ^41, 42^ and careful monitoring of glycemic status is required. Pasireotide-associated hyperglycemia is related to reduced insulin secretion and incretin hormone responses, without changes in hepatic/peripheral insulin sensitivity ^43^. Pasireotide has the highest affinity for SSTR5, which is known to play an important role in mediating insulin secretion. Dipeptidyl peptidase (DPP)-4 inhibitors (e.g. sitagliptin, vildagliptin, saxagliptin, and linagliptin) and glucagon-like peptide-1 (GLP-1) receptor agonists (e.g. liraglutide and exenatide) may be the most effective agents for controlling pasireotide-associated hyperglycemia ^44^.

### Pegvisomant

Pegvisomant is a human GH receptor antagonist which blocks the production of IGF-1. The latest analysis from the ACROSTUDY included 1288 patients and reported IGF-1 control in 63% of them after 5 years on pegvisomant ^45^. Increase in adenoma size was found in 3.2% of the patients and increase in the hepatic enzymes >3 times the ULN in 2.5%; no case of liver failure was described. The current recommendations suggest monitoring liver function tests monthly for the first 6 months and 6-monthly thereafter with consideration of discontinuation of pegvisomant in case of >3-fold elevation of transaminases. As pegvisomant does not have a tumor-suppressive effect, it is proposed that imaging is performed at 6 and 12 months initially and if there is no size change at 1 year, then yearly imaging ^4^.

### Other medical treatments

Dopamine agonists are useful in patients with modest elevations of GH and IGF-1 levels with or without concomitant hyperprolactinemia. A meta-analysis (including 160 patients) estimated that cabergoline as single-agent therapy normalizes IGF-1 levels in 34% of acromegalics and as an add-on therapy to SSAs in 52% of them. This effect may occur even in patients with normoprolactinemia and it is dose independent ^46^.

Clomiphene citrate – a selective estrogen receptor modulator with organ-specific estrogenic and antiestrogenic properties – has been tried as an “add on” therapy together with octreotide LAR and/or cabergoline in a group of 16 male acromegalics with IGF-1 above the ULN for at least 1 year despite the use of available medical therapies. At the end of the study, serum IGF-1 decreased by 41%, leading to normal values in 44% (7/16) of them ^47^.

### Combination of medical treatments

The combination of pegvisomant with cabergoline or SSAs has been reported in two small studies as a potential effective treatment option in patients with acromegaly not responsive to monotherapy. The addition of pegvisomant (10 mg daily for 12 weeks) to cabergoline (offered in a maximum dose of 0.5 mg once daily) reduced significantly the IGF-1 levels in comparison with either cabergoline alone (on a maximum dose of 0.5 mg once daily) or low-dose (10 mg daily) pegvisomant monotherapy; 68% of the patients achieved normal IGF-1 ^48^. Furthermore, the addition of pegvisomant (median dose 60 mg weekly) in partially controlled acromegalics on high-dose SSAs normalized IGF-1 in 57.9% of them ^49^.

## Radiotherapy

Based on the recently published Endocrine Society Clinical Practice Guideline on acromegaly, radiotherapy is a third-line option offered if there is residual tumor mass following surgery and if medical therapy is unavailable, unsuccessful, or not tolerated ^4^.

Modern stereotactic techniques (stereotactic radiosurgery [SRS] or fractionated stereotactic radiotherapy [FSRT]) deliver more localized irradiation and minimize the long-term toxicity. The volume of the target lesion and its proximity to sensitive structures dictate the choice of the radiotherapy technique. Particle radiation delivered as SRS or FSRT has been also applied successfully in the treatment of pituitary adenomas. The physical properties of proton irradiation can offer superior conformality in dose distribution when compared to photons, and the advantage becomes more apparent for large volumes ^50^.

Minniti *et al.* reviewed the results of five studies which used FSRT in 115 patients with acromegaly and showed that at a median follow-up of 54 months (range 28–80), the tumor control rate was 97% ^50^. The 5-year biochemical remission of the disease was 42%. Results of 29 studies including 1215 patients treated with SRS showed that at a median follow-up of 50.6 months (range 6–114 months) tumor control was achieved in 98%. When the 2010 criteria were applied, the 5-year hormonal normalization rate assessed in nine studies which included 528 patients was 43% ^50^.

In a study of 136 acromegalics who received gamma knife (GK) SRS and had a median follow-up 61.5 months, the actuarial remission rates (defined as a normal age- and gender-matched serum IGF-1 level and, in some patients, nadir GH level <1 ng/mL on oral glucose tolerance test, off any medication) at 2, 4, 6, and 8 years post-radiosurgery were 32%, 65%, 73%, and 83%, respectively. New pituitary hormone deficits occurred in 43 patients (32%) and risk factors were a margin dose >25 Gy and tumor volume >2.5 mL ^51, 52^.

Wattson *et al.* assessed the value of proton therapy in 50 patients with acromegaly and reported that the actuarial 3-year remission rate was 26% and the median time until remission was 62 months ^53^.

Cerebrovascular mortality has been found to be increased in patients with pituitary adenoma treated with radiotherapy compared with the general population, and it is related to atherogenesis in the vascular lining from the radiotoxicity ^54^. Risk factors include older age, previous aggressive intracranial surgery, and total dose >45 Gy. The risk for second brain tumors (most commonly meningiomas, gliomas, and chondrosarcomas) in a series of patients irradiated for pituitary adenoma was 2% at 20 years, but another series comparing patients with pituitary adenoma and treated with surgery alone or post-operative radiotherapy did not confirm increased risk ^55, 56^. Furthermore, recent data from a study including 806 patients with a non-functioning pituitary adenoma from the Dutch National Registry of Growth Hormone Treatment in Adults reported that the frequency of secondary intracranial tumors and mortality did not differ between irradiated and non-irradiated subjects ^57^.

## Conclusions

The tools for the management of acromegaly have shown advances in the last few years, offering further perspectives in the treatment of patients with this condition and particularly of those with challenging tumors. A significant amount of literature has been published in recent years, especially on the area of medical treatment, enlightening the pros and cons of the available agents and facilitating our therapeutic decisions. The impact of the currently accepted treatment algorithms on long-term morbidity and mortality remains to be assessed.

## Abbreviations

CI, confidence interval; FSRT, fractionated stereotactic radiotherapy; GH, growth hormone; HR, hazard ratio; IGF-1, insulin growth factor-1; LAR, long-acting release; MRI, magnetic resonance imaging; OOCs, oral octreotide capsules; OR, odds ratio; SMR, standardized mortality ratio; SRL, somatostatin receptor ligand; SRS, stereotactic radiosurgery; SSA, somatostatin analogue; SSTR, somatostatin receptor subtype; ULN, upper limit of normal.
